# Supplementary material for: No evidence for decision fatigue using large-scale field data from healthcare
Source: Commun Psychol. 2025 Feb 26;3:33. doi: 10.1038/s44271-025-00207-8 (PMC11865449; doi:10.1038/s44271-025-00207-8)
Supplement: Supplementary file 2 — Supplementary Material [file 44271_2025_207_MOESM2_ESM.pdf]

communications
psychology

Supplementary Material

Stage 2 Registered Report:
No evidence for decision fatigue using large-scale field data from healthcare

David Andersson[1], Malou Lindberg[2], Gustav Tinghög[1,3], and Emil Persson[1]*

[1] Department of Management and Engineering, Division of Economics, Linköping University, 581 83 Linköping, Sweden

[2] Department of Health, Medicine and Caring Sciences (HMV), Linköping University, 581 83 Linköping, Sweden

[3] Department of Health, Medicine and Caring Sciences (HMV), The National Center for Priority Setting in Health Care, Linköping University, 581 83 Linköping, Sweden

* Corresponding author: Emil Persson (emil.persson@liu.se).

communications
psychology

Supplementary Figures

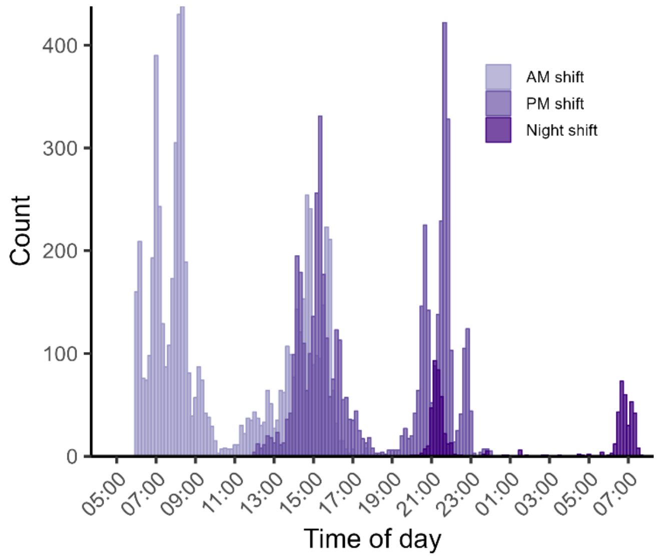

Supplementary Figure 1. First and last call each shift [pilot data]. Substantial overlap in the afternoon between morning and afternoon shifts gives a quasi-experimental setting. [n = 12,442] (This is the same figure as Supplementary Figure 3 in the Stage 1 Supplementary Material, reproduced here for readers' convenience.)

communications
psychology

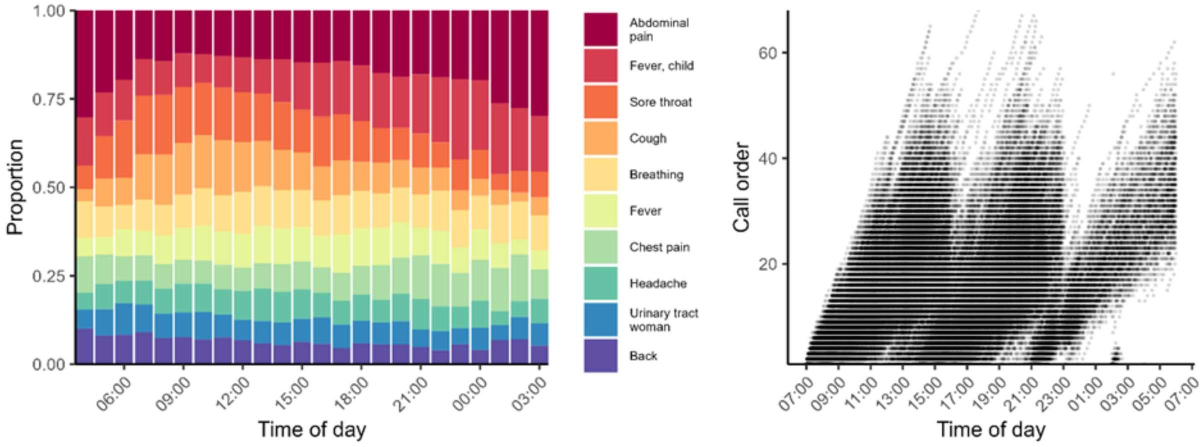

Supplementary Figure 2. Composition of patient types by time of day and call order [pilot data]. ***Left:*** Time distributions are different for different patient types. There are appr. two hundred predefined call categories based on type of medical problem. The ten most common categories are shown in the figure. [n = 37,051] ***Right:*** Call order (ordinal position) is positively correlated with time of day. Each point in the figure represents an individual call indexed by start time (x-axis) and ordinal position among calls handled by the specific nurse during the work shift (y-axis). The characteristic pattern in the figure (three right-tilted funnels) emerges due to the scheduling procedure with three broad shift categories (AM, PM, Night) and staggered start times. [n= 125,886] (This is the same figure as Supplementary Figure 1 in Stage 1 Supplementary Material, reproduced here for readers' convenience.)

communications
psychology

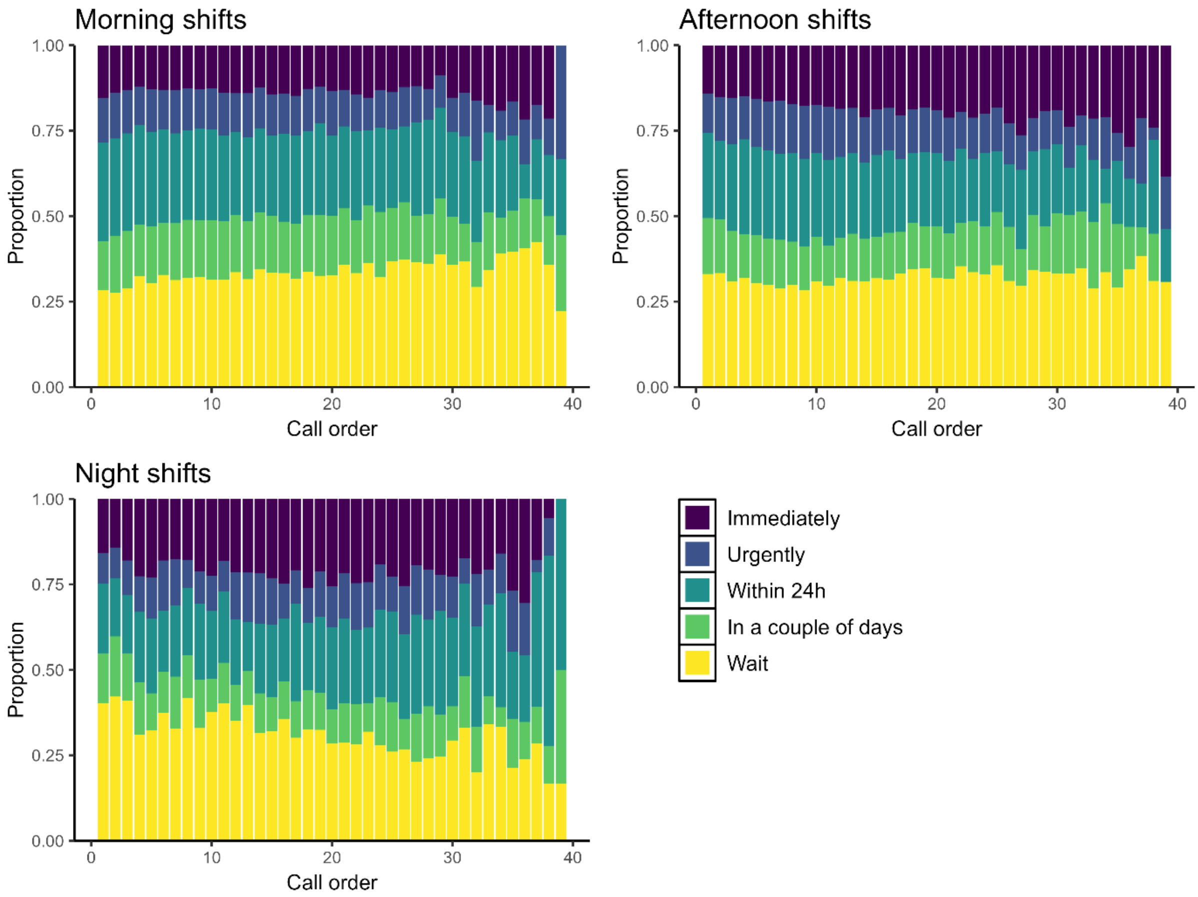

Supplementary Figure 3. Ordinal position of call within a work shift and assigned urgency. Each bar shows the distribution of urgency ratings for calls at that particular ordinal position (call order). Calls in shifts with < 10 calls or at call order > 40 were excluded to focus on shifts that were not part admin and to ensure sufficient number of calls (n = 100) for each bar in the figure. Higher urgency ratings at higher call orders would be expected under decision fatigue. This analysis is not part of our main test battery (for reasons explained in the Methods section and in Supplementary Note 1, below). [Morning, n = 59,231; Afternoon, n = 51,521; Night, n = 9,409]

communications
psychology

Supplementary Tables

Supplementary Table 1. Results for *Consult* and *Call duration*

	Overlap<br>[1]<br>Consult<br>Coef.	Overlap<br>[2]<br>Call duration<br>Coef.	Breaks<br>[3]<br>Consult<br>Coef.	Breaks<br>[4]<br>Call duration<br>Coef.
Morning	-0.031	-14.991		
	(0.066)	(5.759)		
	[-0.159, 0.101]	[-26.637, -4.045]		
Before break			0.046	15.766
			(0.074)	(6.462)
			[-0.096, 0.189]	[3.022, 28.587]
Time 3	0.023	7.784		
	(0.087)	(6.478)		
Time 4	0.109	4.592		
	(0.087)	(6.186)		
Time 5	0.226	-17.522		
	(0.090)	(6.262)		
Time 6	0.233	-8.094		
	(0.095)	(6.483)		
Intercept	-1.371	34.519	-1.592	11.393
	(0.099)	(11.283)	(0.100)	(10.711)
N observations	9,648	9,648	4,940	4,940
N clusters	143	143	77	77
Bayes Factor, BF_{10}	0.007	8.3	0.06	>100

Table notes: In columns [1] and [3], for *Consult*, we used a Bayesian generalized mixed effects model for binary dependent variables, with random intercepts for nurses, and we used weakly informative priors. Standard errors are shown in parentheses. In columns [2] and [4], for *Call duration,* our approach was the same except here we used a Bayesian generalized mixed effects model for continuous dependent variables. Time indicator variables (*Time 2* was reference category) were entered as fixed factors in the overlap sample. 95% credible intervals for estimated coefficients are shown in brackets for the main independent variables. The sample standard deviations for *Call duration* were 307 seconds in the overlap sample and 306 seconds in the breaks sample (can be used to calculate a Cohen's *d* effect size). BF_{10} is the two-sided Bayes Factor for the full model vs. the restricted model that excludes the relevant independent variable (*Morning* in columns [1] and [2] and *Before break* in columns [3] and [4]).

communications
psychology

Supplementary Note 1

We discussed some of our design choices more extensively in the Stage 1 Supplementary Material. Here we reproduced the part about *time of day* and *call order* (why they may not be good fatigue proxies) and we included the relevant figure (above) for readers' convenience.

Designing the study, we used the pilot data to search for the best ways to compare decisions under relatively high vs. low fatigue. In line with the previous literature, we proxy for fatigue using an observational correlate of cumulative mental effort, e.g., time spent at work or since last break, or number of calls taken. In our setting (demanding, repetitive, stressful) it is easy to think of such proxies with plausibly good variation in fatigue. However, the challenge is to find cases where this variation is exogenous, thus not influencing or influenced by relevant outcome variables.

Time of day is an obvious candidate for a fatigue proxy. It is accessible at a low level of data granularity and thus easy to work with, and has often been used in previous studies. However, exploring our data we observed strong temporal patterns in the composition of call types, which also vary in urgency, e.g., chest pain vs. sore throat (Supplementary Figure 2 [in this document], left panel). This means that even if we were to observe changes in decision-making linked to time of day, e.g., higher urgency ratings for calls in the afternoon, we would not know whether this was caused by fatigue or by call composition, or both. For the same reason we would not be able to interpret absence of time patterns in decision-making as evidence against decision fatigue. *Ordinal position of call within a work shift* [i.e., call order] is another candidate but it has the same problem with confounding as time of day, because these variables are strongly correlated (Supplementary Figure 2 [in this document], right panel). We decided to show descriptive results for these two variables because they are so commonly used in the literature, but we do not assign much evidential value to these results and they are not part of our overall assessment of decision fatigue.

communications
psychology

Supplementary Note 2

Index over the most important documents and files for this project: 1) Stage 1 Protocol, 2) Stage 1 Supplementary Material, 3) Stage 2 manuscript (main article file), 4) Stage 2 Supplementary Material (this document), 5) Data and code to reproduce the study's main analyses (both for the pilot data and target data), 6) Data handling choices, and 7) Scripts for power analysis and sensitivity power analysis.

Note that the title changed between Stage 1 and Stage 2, from "A strong test of decision fatigue using large-scale field data from healthcare" to "No evidence for decision fatigue using large-scale field data from healthcare."

All these files can be accessed via the project's OSF repository: https://osf.io/8tvfs/ (Per Journal policy, some or all of these files may also be placed on other repositories, but the relevant files will be the same.)
