# Supplementary material for: No evidence for decision fatigue using large-scale field data from healthcare
Source: Commun Psychol. 2025 Feb 26;3:33. doi: 10.1038/s44271-025-00207-8 (PMC11865449; doi:10.1038/s44271-025-00207-8)
Supplement: Supplementary file 3 — Reporting Summary [file 44271_2025_207_MOESM3_ESM.pdf]

Corresponding author(s): *Emil Persson*

Last updated by author(s): *Jan 30, 2025*

Reporting Summary

Nature Portfolio wishes to improve the reproducibility of the work that we publish. This form provides structure for consistency and transparency in reporting. For further information on Nature Portfolio policies, see our Editorial Policies and the Editorial Policy Checklist.

Statistics

For all statistical analyses, confirm that the following items are present in the figure legend, table legend, main text, or Methods section.

n/a	Confirmed	
☐	☒	The exact sample size (*n*) for each experimental group/condition, given as a discrete number and unit of measurement
☐	☒	A statement on whether measurements were taken from distinct samples or whether the same sample was measured repeatedly
☐	☒	The statistical test(s) used AND whether they are one- or two-sided<br>*Only common tests should be described solely by name; describe more complex techniques in the Methods section.*
☐	☒	A description of all covariates tested
☒	☐	A description of any assumptions or corrections, such as tests of normality and adjustment for multiple comparisons
☐	☒	A full description of the statistical parameters including central tendency (e.g. means) or other basic estimates (e.g. regression coefficient) AND variation (e.g. standard deviation) or associated estimates of uncertainty (e.g. confidence intervals)
☒	☐	For null hypothesis testing, the test statistic (e.g. *F*, *t*, *r*) with confidence intervals, effect sizes, degrees of freedom and *P* value noted<br>*Give P values as exact values whenever suitable.*
☐	☒	For Bayesian analysis, information on the choice of priors and Markov chain Monte Carlo settings
☒	☐	For hierarchical and complex designs, identification of the appropriate level for tests and full reporting of outcomes
☐	☒	Estimates of effect sizes (e.g. Cohen's *d*, Pearson's *r*), indicating how they were calculated

Our web collection on statistics for biologists contains articles on many of the points above.

Software and code

Policy information about availability of computer code

Data collection	*No software was used.*
Data analysis	*The statistical software R version 4.3.3 was used.*

For manuscripts utilizing custom algorithms or software that are central to the research but not yet described in published literature, software must be made available to editors and reviewers. We strongly encourage code deposition in a community repository (e.g. GitHub). See the Nature Portfolio guidelines for submitting code & software for further information.

Data

Policy information about availability of data

All manuscripts must include a data availability statement. This statement should provide the following information, where applicable:

- Accession codes, unique identifiers, or web links for publicly available datasets
- A description of any restrictions on data availability
- For clinical datasets or third party data, please ensure that the statement adheres to our policy

All data needed to reproduce the study's main analyses (main tests and evaluation) is publicly available on the project's OSF repository at https://osf.io/8tvfs/. These data contain all variables and all observations (at full disaggregation but without participant identifiers) used in the main analyses for each of the two analysis subsets, overlap and breaks respectively. For ethical reasons (participant integrity), data and features not used in the planned final analyses are deleted before

uploading, and there is no way for readers to access the full raw data. Data from the pilot study (same structure) is available on the same OSF repository (stage 1 folder).

Human research participants

Policy information about studies involving human research participants and Sex and Gender in Research.

Reporting on sex and gender	*This information was not collected.*
Population characteristics	*This information was not collected. Research participants are specialized nurses working at a national telephone medical advice and triage service. Main focus for the research are their decisions, and this data was obtained as registry data.*
Recruitment	*n/a. See above (registry data) and the manuscript for further sampling considerations.*
Ethics oversight	*Swedish Ethical Review Authority*

Note that full information on the approval of the study protocol must also be provided in the manuscript.

Field-specific reporting

Please select the one below that is the best fit for your research. If you are not sure, read the appropriate sections before making your selection.

☐ Life sciences ☒ Behavioural & social sciences ☐ Ecological, evolutionary & environmental sciences

For a reference copy of the document with all sections, see nature.com/documents/nr-reporting-summary-flat.pdf

Behavioural & social sciences study design

All studies must disclose on these points even when the disclosure is negative.

Study description	*Quantitative data. Registered report using behavioral data, obtained as registry data, in two steps; first pilot data then protocol registration (analysis plan and sampling i.e. scope of new batch of registry data etc fixed), then target data and analyses as planned. Approach/design is retrospective observational, but planned in advance.*
Research sample	*Healthcare professionals (specialized nurses) working at a national telephone triage and medical advice service in Sweden.*
Sampling strategy	*Two stages, obtained behavioral data from registry for fix time period. Each time subsequently excluded data (second time preregistered, see above) to arrive at relevant analysis subsamples tailored for our overall analytic approach.*
Data collection	*Behavioral data obtained as registry data.*
Timing	*Nov 2021, received Stage 1 data (pilot data) [from registry, time period ca Oct--Dec 2019 and Oct--Dec 2020] that we had previously asked for; May 7, 2024, protocol accepted in principle, May 13, 2024, sent first formal request to data owner (registry) for access to target data, Sept 24, 2024 target data (i.e., Stage 2 data) delivered [from registry, time period ca Sept/Oct--Dec 2021 and Sept/Oct--Dec 2022].*
Data exclusions	*Yes, to arrive at relevant analysis subsamples. Used detailed, pre-established exclusion criteria for the target data. See manuscript for details.*
Non-participation	*n/a*
Randomization	*Registry data. Design was retrospective observational but planned in advance, and partly quasi-experimental, where analysis subsamples ("overlap sample", see paper) could plausibly be regarded as near-random allocation to comparison groups due to existing variation in scheduling.*

Reporting for specific materials, systems and methods

We require information from authors about some types of materials, experimental systems and methods used in many studies. Here, indicate whether each material, system or method listed is relevant to your study. If you are not sure if a list item applies to your research, read the appropriate section before selecting a response.

Materials & experimental systems

n/a	Involved in the study
☒	☐ Antibodies
☒	☐ Eukaryotic cell lines
☒	☐ Palaeontology and archaeology
☒	☐ Animals and other organisms
☒	☐ Clinical data
☒	☐ Dual use research of concern

Methods

n/a	Involved in the study
☒	☐ ChIP-seq
☒	☐ Flow cytometry
☒	☐ MRI-based neuroimaging
